# Development and Characterization of Polymer Blends Based on Polyvinyl Alcohol for Application as Pharmaceutical Dosage Form

**DOI:** 10.3390/polym17162203

**Published:** 2025-08-12

**Authors:** Zarina A. Kenessova, Grigoriy A. Mun, Perizat I. Urkimbayeva, Assel K. Toktabayeva, Raikhan K. Rakhmetullayeva, Bayana B. Yermukhambetova, Zhazira Kenzhebai, Zhuldyzay T. Kurmanova, Mubarak Yermaganbetov, Adilet Zh. Alikulov

**Affiliations:** 1 Department of Chemistry and Technology of Organic Substances, Natural Compounds and Polymers, Faculty of Chemistry and Chemical Technology, Al-Farabi Kazakh National University, Almaty 050040, Kazakhstan; zarina.kenesova@gmail.com (Z.A.K.); mungrig@yandex.ru (G.A.M.); raikhan.rakhmetullayeva@gmail.com (R.K.R.); baya_yerm@mail.ru (B.B.Y.); kurmanovazhuldyzay@gmail.com (Z.T.K.); alikulov.adilet@gmail.com (A.Z.A.); 2 National Engineering Academy of the Republic of Kazakhstan, Almaty 050060, Kazakhstan; 3 Department of Chemical and Biochemical Engineering, Satbayev University, Almaty 050013, Kazakhstan

**Keywords:** polyvinyl alcohol, methylcellulose, hydrogels, active pharmaceutical ingredient, mucoadhesive properties

## Abstract

Mixtures containing polyvinyl alcohol (PVA) and methylcellulose (MC) were obtained and used to synthesize hydrogels in various ratios of components. The swelling kinetics of the resulting hydrogels were studied, revealing that the equilibrium swelling degree in artificial saliva is nearly twice as high as in water. It was found that increasing the volumetric content of PVA in the mixture leads to a higher swelling degree. The kinetics of active pharmaceutical ingredient (API) sorption and release from the hydrogels were also investigated. It was demonstrated that hydrogels with a higher PVA content exhibit greater sorption capacity; however, the release of the API from such samples occurs at a slower rate. For the first time, the mucoadhesive properties of PVA-MC-based hydrogels were studied. It was established that the PVA-MC hydrogel with a ratio of 6:4 vol.% remained on the surface of the porcine cheek mucosa for two days, the 5.5:4.5 vol.% sample detached after 24 h, and the 5:5 vol.% sample adhered for approximately 10 h. These findings confirm the mucoadhesive potential of the hydrogels and their suitability for buccal drug delivery forms. The synthesized PVA-MC hydrogels are promising for applications in medicine and pharmacology.

## 1. Introduction

In the modern pharmaceutical industry, the search for effective and safe drug delivery systems remains a key area of research. Polymer-based dosage forms represent delivery systems in which polymers serve as carriers or matrices for active pharmaceutical ingredients (APIs). This is a crucial aspect of contemporary pharmaceutics, as such forms can enhance bioavailability, control drug release, enable targeted delivery, and reduce undesirable side effects. A wide range of polymers is used in the pharmaceutical industry and scientific research to develop various dosage forms [1].

A number of unique properties of polymeric hydrogels—such as high water content, tunable mechanical strength, biocompatibility, and the ability to respond to environmental stimuli—make them particularly attractive for specific applications in medicine and pharmacy [2]. However, the challenge of developing a polymer hydrogel that meets a broad spectrum of requirements while also being accessible, low cost, and easy to produce remains relevant. In this context, the use of PVA as the basis for polymeric hydrogels appears highly promising. Polyvinyl alcohol (PVA) is a high-tonnage synthetic polymer known for its biocompatibility and hydrophilicity [3].

PVA is a water-soluble synthetic polymer widely used in various fields due to its unique properties such as excellent chemical resistance, biocompatibility, and film-forming ability. The production of polyvinyl alcohol begins with the polymerization of vinyl acetate, which is the monomer precursor of PVA. This monomer undergoes polymerization in the presence of initiators, forming polyvinyl acetate. Subsequently, polyvinyl acetate is subjected to hydrolysis (saponification) in aqueous or alcoholic solution using either an acidic or alkaline catalyst. As a result of hydrolysis, acetate groups are removed, forming polyvinyl alcohol. The degree of hydrolysis can be varied, allowing for control over the properties of the final product [4,5,6,7].

The degree of hydrolysis, molecular weight, and crystallinity significantly influence the water solubility and physical properties of PVA, including its film-forming capability. PVA generally has a partially crystalline structure and is characterized by properties such as chemical resistance, water solubility, and biodegradability. The similarity of its physical properties to human tissues makes it biocompatible, allowing for interaction with protein molecules while minimizing cellular adhesion and avoiding toxic effects.

Fully hydrolyzed forms can form PVA hydrogels with tunable properties through the cross-linking of linear polymers, ultimately resulting in the formation of polymeric hydrogels [8]. PVA-based hydrogels can be synthesized using low-molecular-weight bifunctional cross-linking agents such as glutaraldehyde. Another cross-linking method involves gamma irradiation, while a third widely used approach is based on freeze–thaw cycling, which promotes the formation of physical cross-links between polymer chains.

Radiation cross-linking involves the use of high-energy rays such as gamma rays, electron beams, and X-rays for the direct irradiation of PVA solutions. When PVA is exposed to high-energy radiation, macromolecular free radicals are formed due to the direct effect of radiation and the influence of active radiolysis particles of water. These free radicals can be located on secondary and tertiary carbon atoms, respectively. Two such radicals can couple through a double group bonding reaction, forming cross-linking bonds. As the number of cross-links increases, gelation begins, gradually forming a three-dimensional network structure in the system [9,10,11,12]. PVA hydrogels obtained through irradiation do not require cross-linking agents, which ensures high purity and good optical transparency. However, the high-energy reaction conditions may lead to a deterioration of mechanical properties and other material characteristics. The advantage of such hydrogels lies in the speed of the reaction, which can occur at room temperature and atmospheric pressure [13]. However, due to the intensity of the radiation, not all materials can be used in hydrogel systems.

These features of radiation-cross-linked PVA hydrogels are particularly valuable in biomedical applications, where sterility, biocompatibility, and the absence of toxic residues are critical. Due to their high purity, moisture retention capacity, structural stability, and tunable permeability, such hydrogels meet modern requirements for biomedical polymeric materials, including bioinertness, biocompatibility, and potential biodegradability [14]. PVA-based hydrogels are widely utilized in controlled drug delivery systems, microcapsules, wound dressings, artificial organ and tissue constructs, as well as in smart materials that respond to external stimuli such as temperature and pH [15].

PVA-based hydrogels are widely used as drug carriers, incorporating small-molecule therapeutics to enhance their stability. Due to the environmental resistance of PVA, the drug release mechanism from PVA hydrogels typically follows a zero-order kinetics model, in which the release rate of the active compound remains constant over time, independent of its concentration [16].

For instance, in PVA hydrogel systems loaded with analgesic agents, it has been demonstrated that the material provides sustained drug release over an extended period without the initial burst effect commonly observed in other polymer-based carriers [17]. This is particularly important for the development of implantable and transdermal drug delivery systems, where stable pharmacokinetics and minimal side effects are essential.

Moreover, the structural tunability of PVA hydrogels—achieved by incorporating nanoparticles, other polymers, or functional additives—allows for precise control over system properties such as diffusion rate, degradation profile, and responsiveness to external stimuli [15]. Such multifunctional composites are especially promising for the development of smart drug delivery systems capable of releasing therapeutic agents in response to pH changes, temperature fluctuations, enzymatic activity, or other physiological triggers [18].

PVA exhibits superior biomimetic properties compared to many other synthetic polymers, such as high hydrophilicity, flexibility, softness, and the ability to form porous, gel-like structures that mimic the extracellular matrix. These characteristics make it highly suitable for applications in tissue engineering and as a platform for controlled drug delivery systems [19,20,21,22]. PVA-based hydrogels can serve as scaffolds for various cell types, including pancreatic and corneal cells, supporting cell implantation without eliciting significant immune responses [23].

Blending PVA with other polymers can significantly improve its mechanical performance. For example, incorporating chitosan (CT) into PVA hydrogels enhances tensile strength, elasticity, and surface hydrophilicity while also improving the uniformity of the network structure, reducing porosity, and increasing bioabsorbability, features essential for preserving cell viability [24]. When mixed with polyethylene glycol (PEG), PVA can enable controlled drug release, with increased PEG content reducing hydrogel swelling. Modifications using cellulose powder and PEG further improve biocompatibility and cytocompatibility, facilitating applications in cell culture and tissue regeneration [25].

PVA microparticles have also been used to encapsulate drugs via membrane emulsification and chemical cross-linking, allowing for higher drug loading efficiency. Furthermore, incorporating silver nanoparticles into PVA enhances its antibacterial properties, making it useful for wound dressings [26]. However, it is important to note that blending PVA with certain polymers may sometimes introduce toxicity, particularly when chemical cross-linking is involved [27].

In PEG/PVA hydrogel systems, drugs such as aspirin demonstrate sustained release profiles across a range of pH-buffered environments [28]. Such systems are being investigated for localized drug delivery and targeted cancer therapies. Moreover, in the context of diabetes—a metabolic disorder marked by impaired glucose, lipid, and protein metabolisms due to insufficient insulin production and islet cell dysfunction—PVA hydrogels modified with glycerol have shown promise for prolonged insulin release [29,30]. These PVA/PEG-based hydrogels also exhibit favorable physical and chemical characteristics for pH-responsive drug delivery and wound healing applications.

Mixtures and complexes containing polyvinyl alcohol are widely used in pharmacology and medicine, which contributes to conducting in-depth studies of their various characteristics and to the creation of combinations based on such polymers with different compounds [31]. Hydrogels based on polyvinyl alcohol are examples of promising polymeric materials. They are biocompatible, which is a very important criterion for their use in medicine, since they are non-toxic, non-carcinogenic, non-allergenic, and non-mutagenic, and, in addition, they are mechanically strong and demonstrate resistance under various external changes. Taking into account such characteristics of polymeric materials based on polyvinyl alcohol, the development of various bases for pharmaceutical dosage forms and drug carriers is relevant, since prolonging the activity of a drug and reducing its toxicity is currently a promising approach in pharmaceuticals.

The aim of this work is to obtain mixtures and complexes containing polyvinyl alcohol as a base for pharmaceutical dosage forms.

## 2. Materials and Methods

### 2.1. Characteristics of the Starting Materials

PVA—a product of Merck KGaA (Darmstadt, Germany)—has a molecular weight of 145,000, a degree of hydrolysis of 98%, and is used without further processing. Methylcellulose—a product of Sigma-Aldrich (St. Louis, MO 63178, USA)—has an average molecular weight of 14,000, a degree of substitution of OCH_3_ groups γ = 1.7, and a viscosity of a 2% aqueous solution of 0.015 Pa·s and is used without additional purification. Ceftriaxone solutions (OJSC BZMP, Minsk, Republic of Belarus) of various concentrations were prepared by dissolving the powder of the active substance intended for the preparation of an injectable solution with a dosage form of 1 g. Calcium chloride (CaCl_2_), sodium bicarbonate (NaHCO_3_), disodium hydrogen phosphate (Na_2_HPO_4_), and hydrochloric acid (HCl), manufactured by ReaktivSnab (Ufa, Russia), were used without additional purification. Distilled water was used for the preparation of the solutions.

### 2.2. Preparation of PVA-MC-Based Mixtures for the Production of Radiation Cross-Linked Hydrogels

An aqueous solution of PVA was prepared by dissolving 8 g PVA granules in 100 mL of distilled water at 80 °C with constant mechanical stirring until a clear solution was obtained. An aqueous solution of MC was prepared at the same concentration as the PVA solution. In total, 8 g MC was dissolved in 50 mL of hot water (80–90 °C), and then the solution was cooled to room temperature. After that, 50 mL of cold water was added, and the solution was left in a refrigerator for 10–12 h for complete dissolution. The 8% aqueous PVA solution was then added to the 8% aqueous MC solution and stirred using a magnetic stirrer. The resulting solution was poured into 10 mL polyethylene containers. The polyethylene containers were hermetically sealed and then sent to the ILU-10 facility for radiation exposure. All solutions were irradiated with an electron beam at a dose of 75 kGy under ambient conditions. The resulting hydrogels were then dried at room temperature and sent for further characterization. PVA-MC mixtures were prepared in the following volume ratios: φ_PVA_:φ_MC_ = 6:4; 5.5:4.5; and 5:5 vol.%.

Radiation exposure of the obtained mixture was carried out at the ILU-10 electron accelerator, where the containers with the mixture passed through the beam of accelerated electrons. On a conveyor belt, the samples moved under the radiation source for a certain period. For each pass of the samples, the operator had the ability to set the required parameters of the accelerator. The radiation dose per pass is 25 kGy. The total exposure dose of the sample is 75 kGy (3 passes).

### 2.3. Physicochemical Research Methods

IR spectroscopic analysis was performed using an ALPHA FT-IR spectrometer (Bruker, Ettlingen, Germany). Measurements were carried out in the range of 500–4000 cm^−1^. The attenuated total reflectance (ATR) method was used in this study. Solutions of the PVA-MC mixture were poured onto glass plates at room temperature, resulting in the formation of films. The resulting gel films were then applied to the ATR element and lightly pressed.

The swelling degree of the hydrogels was determined by the gravimetric method. The swelling degree study was carried out in two media: in water and in artificial saliva solution.

Hydrogel samples weighing 0.1 g were placed in 30 mL of distilled water/artificial saliva, and the mass of the hydrogel samples was measured using analytical balances every 10, 20, and 30 min. The swelling degree was calculated using Equation (1):

(1) $$\alpha \;=\;\frac{m_{t}-m_{0}}{m_{0}\;}$$

where *m*_0_ is the mass of the dry sample in grams, and *m_t_* is the mass of the swollen sample in grams at a given time point.

The kinetics of sorption and release of the API by the hydrogel were studied using a UV spectrophotometer SPECORD^®^ 200 PLUS (Analytik Jena GmbH+Co. KG, Jena, Germany).

To study the sorption process, a sample weighing 0.1 g was immersed in a ceftriaxone solution (10 mg/mL) for 12 h. The drug absorption was measured every hour, taking into account the change in the optical density of the ceftriaxone solution at a wavelength of λ = 293 nm, using Equation (2):

(2) $$Q_{e}\;=\;\frac{\left(C_{0}-C_{e}\right)\cdot V}{m}$$

where *Q_e_* is the amount of sorbed substance per unit mass of hydrogel, *C*_0_ is the initial concentration of the substance in the solution, *C_e_* is the equilibrium concentration of the substance in the solution after sorption, *V* is the volume of the solution, and *m* is the mass of the hydrogel.

The absorption was converted into the drug concentration using a linear calibration curve, and then the cumulative percentage of released ceftriaxone was calculated using a dilution factor. Measurements were performed in a quartz cuvette with a path length of 1 mm.

The kinetics of drug release was determined by immersing hydrogels with immobilized API in physiological saline solution (0.9% NaCl) for 12 h and measuring the optical density of the solution every hour. All experiments were conducted three times, and based on the collected data, graphs were constructed to illustrate the concentration dependence of optical density. A calibration curve was used to calculate the concentrations of sorbed and released drug substances.

To study the release kinetics of the API from hydrogels based on PVA-MC mixtures, samples in the form of discs with a diameter of 10–12 mm and a thickness of 0.2 mm were prepared. The samples were placed in 10 mL of physiological solution at a temperature of 36 °C. This approach is widely used in in vitro studies to simulate physiological conditions close to in vivo environments. For example, a study published in Acta Pharmacologica Sinica describes the use of phosphate-buffered saline (PBS) at a temperature of 37 °C to evaluate the release of pharmaceutical substances from polymer hydrogels, confirming the relevance of such conditions for modeling the internal environment of the human body [32]. The release of the API was determined using a calibration graph representing the dependence of the optical density of the API on its concentration. The ceftriaxone content in the solution was determined using the SPECORD^®^ 200 PLUS spectrophotometer (Analytik Jena GmbH+Co. KG, Jena, Germany) at the absorption maximum in the UV region at λ = 293 nm.

During the experiment, the contents of the cell were continuously stirred using a magnetic stirrer, and at predetermined time intervals, samples of the solution were taken to determine the ceftriaxone content. After each measurement, the sample was returned to the cell. The amount of API released over time *t* was calculated using the calibration line according to Equation (3):

(3) $$W\;=\;\frac{C}{C_{0}}\;\times \;100\%$$

where *W* is the amount of released API, *C* is the concentration of ceftriaxone in the surrounding solution at time *t*, and *C*_0_ is the initial concentration of ceftriaxone in the starting solution. The amount of released ceftriaxone was determined relative to the initial concentration in the polycomplex composition. Here, 100% was considered the amount of API that should pass through the membrane upon reaching equilibrium.

The antibacterial properties of PVA-MC hydrogels with ceftriaxone against conditionally pathogenic bacteria *Staphylococcus (S.) aureus* and *Erwinia (E.) carotovora* were investigated using the disk diffusion method on solid nutrient media. Each of the hydrogel samples with ceftriaxone, a control hydrogel sample without antibiotic, and a control paper disk with 1% Metrogyl Denta were placed on an experimental agar plate inoculated with a suspension of *S. aureus* and *E. carotovora* (0.5 McFarland).

The inoculation procedure was performed as follows: a sterile cotton swab was immersed in a standard microbial suspension; then, the excess inoculum was removed by pressing the swab against the wall of the test tube. The inoculation was carried out using streaking movements in three directions, rotating the Petri dish by 60 °C after each pass. Using sterile forceps, three hydrogel samples weighing approximately 2 g each were cut and placed on the surface of the medium and then gently pressed with forceps to ensure full contact with the agar. Immediately after sample application, the Petri dishes were placed upside down in a thermostat and incubated at 35 °C for 24 h.

The results were evaluated visually by assessing the presence or absence of test strain growth in the experimental zones.

The mucoadhesive properties of radiation-cross-linked PVA-MC hydrogels were evaluated using the “rotating basket” method, by measuring the residence time of dried hydrogels on the surface of porcine cheek mucosa. Samples of radiation-cross-linked hydrogels were fixed onto the mucosal surface of the porcine cheek and immersed in a vessel containing artificial saliva solution. The experiment was conducted at 37 °C, with a stirring speed of 80 rpm, and was repeated three times. The adhesion of hydrogels to the mucosal surface was assessed visually until detachment of the hydrogels occurred.

## 3. Results and Discussion

### 3.1. Preparation of Mixtures Based on PVA-MC

Chemical cross-linking remains the most common method for hydrogel formation to date, and the properties of the resulting hydrogel depend on the concentration of the monomer, the cross-linking agent, and the reaction conditions. Mixtures based on PVA have been developed using chemical cross-linking with glutaraldehyde. Glutaraldehyde is frequently used as a cross-linking agent for producing PVA hydrogels due to its ability to form strong covalent bonds between polymer chains. However, not all cross-linking agents are safe for pharmaceutical use. For instance, although glutaraldehyde is widely used for chemical cross-linking, it exhibits cytotoxicity, which limits its application in biomedical areas such as drug carriers and implants. In contrast to chemical cross-linking, the method of radiation cross-linking does not require the addition of potentially toxic chemical agents, which makes the resulting hydrogels more biocompatible and safe for medical use. In addition, radiation exposure allows for more precise control over the degree of cross-linking by adjusting the irradiation dose. This enables the production of hydrogels with the desired mechanical and physical properties without changing the reagent composition. The radiation cross-linking process also facilitates more uniform distribution of cross-links throughout the hydrogel volume, ensuring a homogeneous structure and material properties. Thus, radiation cross-linking represents a preferred method for obtaining biocompatible, pure, and property-controlled materials, particularly for biomedical applications.

**Figure 1:**
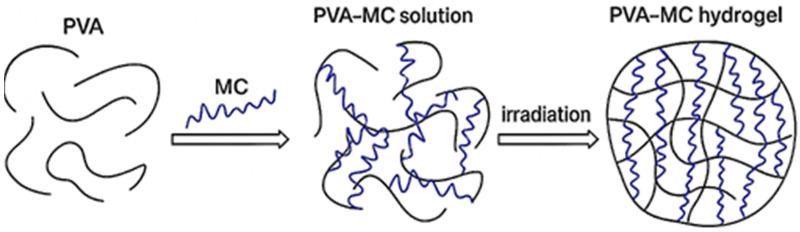
Schematic representation of the process of obtaining PVA-MC-based hydrogels irradiated with an electron beam.

Figure 1 presents a schematic illustration of the process of producing PVA-MC-based hydrogels irradiated with an electron beam. The mechanism of the electron-beam-induced cross-linking reaction of hydrogels is as follows: when aqueous polymer solutions are irradiated with an electron beam, a high dose of energy is absorbed, leading to the formation of a significant number of macroradicals. These macroradicals then participate in radical recombination reactions. As a result of these reactions, new cross-linked bonds form between the macroradicals of the polymers, leading to the formation of a more stabilized and compact hydrogel structure.

**Figure 2:**
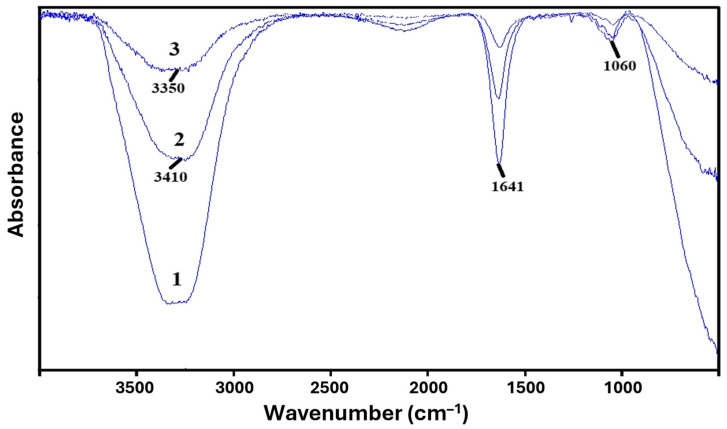
FTIR spectra of PVA-MC-based hydrogels: φ_PVA_:φ_MC_ = 6:4 (1); 5.5:4.5 (2); 5:5 vol.% (3).

For the obtained PVA-MC-based hydrogels, FTIR spectroscopic analysis was carried out on the samples. In the FTIR spectra of PVA-MC (Figure 2), intense stretching vibrations are observed in the region of 1051–1060 cm^−1^, which are characteristic of ether C–O–C groups of the anhydroglucose ring in the polysaccharide structure, as well as stretching vibrations in the region of 1641 cm^−1^, corresponding to carbonyl C–O vibrations of glucose in MC.

**Figure 3:**
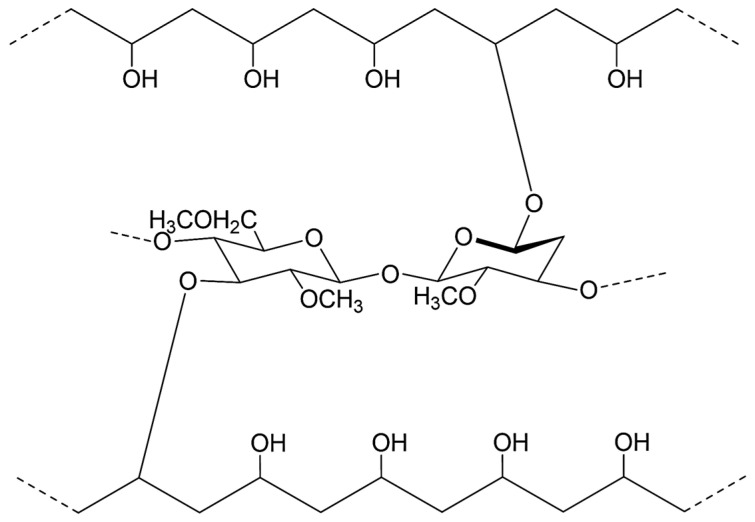
Proposed scheme of interaction between PVA and MC.

The stretching vibrations of the hydroxyl (–OH) groups of methylcellulose are observed in the region of 3448 cm^−1^. The broad peak around 3350 cm^−1^ corresponds to the hydroxyl –OH group of PVA. The detected absorption bands correspond to the results reported in the literature [33,34,35]. Thus, the spectra show characteristic bands corresponding to the functional groups of both PVA and MC, indicating interaction between the macromolecules of the given polymers (Figure 3).

### 3.2. Study of Basic Physico-Chemical Properties of PVA-MC-Based Hydrogels

Swelling of hydrogels is a key phenomenon that determines their physicochemical and biological properties. Swelling is characterized by the hydrogel’s ability to absorb water or biological fluids, thereby increasing in volume. This process occurs due to the hydrophilic groups in the polymer network that interact with water molecules. The main factors influencing the degree of swelling are the chemical composition of the polymer, the cross-linking density, and the external environment (pH, temperature, and ionic strength). Polymers with a large number of hydrophilic groups (e.g., hydroxyl, carboxyl, and amide groups) have a high swelling capacity. The higher the degree of polymer cross-linking, the less space there is for water to penetrate, which reduces the swelling degree. Conditions such as pH and temperature can significantly affect the swelling process. For example, polymers containing acidic or basic groups may vary in their degree of swelling depending on the pH of the medium. The network structure of the cross-linked hydrogel allows solution molecules to penetrate into the polymer mesh via diffusion, which leads to an increase in the mass and volume of the samples while maintaining their shape. Swelling continues until the hydrogel reaches an equilibrium state [36,37,38,39,40].

**Figure 4:**
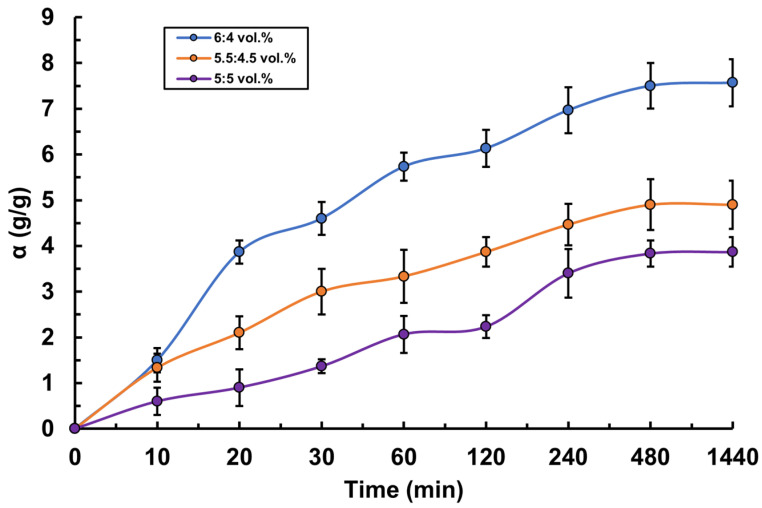
Swelling kinetics of PVA-MC-based hydrogels in water: φ_PVA_:φ_MC_ = 6:4; 5.5:4.5; 5:5 vol.%.

**Figure 5:**
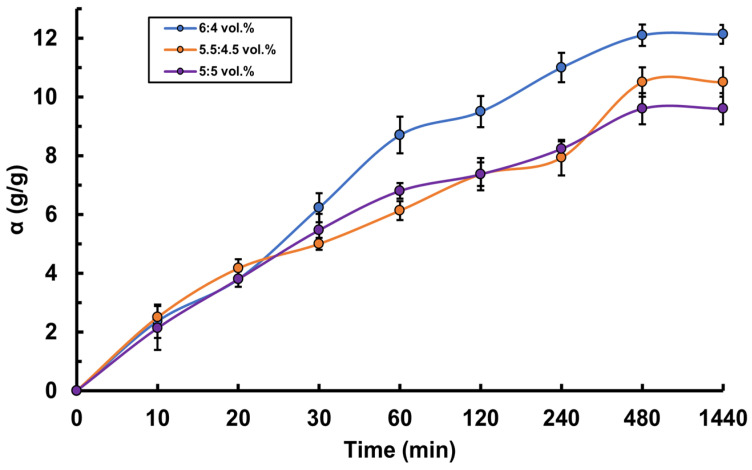
Swelling kinetics of PVA–MC-based hydrogels in artificial saliva: φ_PVA_:φ_MC_ = 6:4; 5.5:4.5; 5:5 vol.%.

For the hydrogels based on PVA-MC mixtures of various compositions obtained in this study, the swelling kinetics were investigated. The study of the swelling degree was carried out in two media: in water and in artificial saliva solution. It was established that hydrogels with a higher concentration of PVA demonstrated a greater degree of swelling (Figure 4). For example, in both solutions, the hydrogel with a PVA-MC ratio of 6:4 vol.% shows the maximum equilibrium swelling degree. In water, it reaches 7.5 g/g, and in artificial saliva 12.13 g/g, which is higher than that of the other hydrogels. This is due to the fact that PVA possesses high hydrophilicity, which contributes to increased water absorption and swelling of the polymer network. The interaction between PVA and MC after radiation cross-linking creates a structure capable of effectively retaining water. It was also found that the swelling degree of the hydrogels in artificial saliva solution is higher than in water (Figure 5). This pattern was observed for all hydrogel ratios. For instance, for the ratio φ_PVA_:φ_MC_ = 5.5:4.5 vol.%, the maximum equilibrium swelling degree in water is 5 g/g, while in artificial saliva, it is 10.5 g/g, which is twice as high. This phenomenon can be explained by several factors related to the composition and properties of artificial saliva compared to pure water. The artificial saliva solution contains various electrolytes and salts, such as sodium, calcium, and phosphates. These ions may interact with the functional groups of the polymers in the hydrogel, such as the hydroxyl groups in PVA and methoxy groups in MC, enhancing the swelling through ion exchange and the formation of additional intermolecular bonds. Moreover, the artificial saliva solution may have higher osmotic pressure compared to pure water. This difference contributes to a more active influx of water into the hydrogel, increasing its swelling capacity. During the first 8 h, a significant increase in the swelling degree of PVA-MC hydrogels is observed in both media, indicating rapid solvent molecule absorption by the hydrogel, followed by a gradual approach to a plateau.

In recent years, there has been a significant increase in interest in health-related issues, driven by longer life expectancy and improvements in national well-being. The pharmaceutical industry has greatly contributed to this growing interest through innovation, improved drug accessibility, preventive measures, and integration with digital technologies. These efforts help people live longer and healthier lives by providing access to advanced methods of treatment and disease prevention. The continuous development of new drugs for the treatment of chronic diseases, infectious illnesses, and cancer significantly improves both the quality and duration of life. The development of new formulations and drug delivery methods, such as sustained release or targeted delivery, enhances therapeutic effectiveness and reduces side effects. Understanding the kinetics of drug release from hydrogels allows for the design of delivery systems that provide gradual and sustained release of the API into the body, maintaining its therapeutic concentration at the required level for a defined period of time. Through controlled release, it is possible to reduce the dosing frequency and lower the risk of toxic effects associated with sharp fluctuations in API concentration in the bloodstream [37].

This study includes an analysis of the potential use of PVA-MC-based hydrogels in various ratios as drug delivery vehicles. For this purpose, the kinetics of sorption and release of the pharmaceutical compound from the hydrogel were studied. Ceftriaxone was used as the model drug compound. Ceftriaxone is a broad-spectrum antibiotic belonging to the class of third-generation cephalosporins. It is effective against a wide range of Gram-positive and Gram-negative bacteria, making it suitable for the treatment of various infections.

Ceftriaxone is a semi-synthetic antibiotic containing a β-lactam ring, which is the key structural component responsible for its antibacterial activity. It inhibits the bacterial cell wall synthesis, leading to bacterial death. It is effective against *Streptococcus pneumoniae*, *Escherichia coli*, *Neisseria gonorrhoeae*, and many other bacteria.

**Figure 6:**
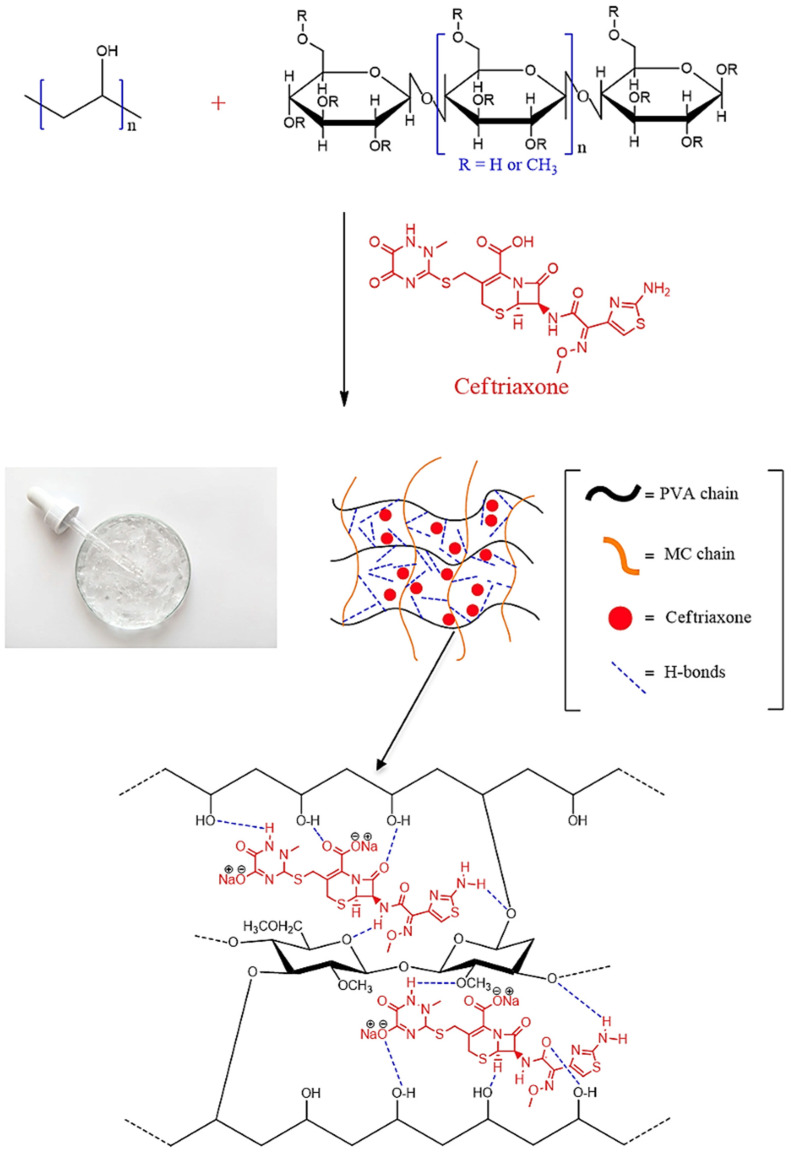
Scheme of interaction between the PVA-MC hydrogel and the API.

Since ceftriaxone possesses a broad spectrum of antibacterial activity and is used in the treatment of skin wounds, the given hydrogel shows potential as a wound dressing material. The penetration of the drug into the PVA-MC-based cross-linked polymer network is illustrated in the proposed scheme (Figure 6).

Upon immobilization of ceftriaxone within the PVA-MC-based hydrogel, the following types of interactions may occur between them:

1. Hydrogen bonding: Hydroxyl groups (–OH) in the structure of PVA and MC can form hydrogen bonds with the carboxyl (–COOH) and amide (–CONH_2_) groups of ceftriaxone.
2. Van der Waals forces: Non-covalent intermolecular interactions arising from the interaction of dipole (or multipole) moments of molecules and the polarization of their electron clouds.
3. Hydrophobic interactions: Between the non-polar parts of the ceftriaxone molecules and the polymer matrix.

**Figure 7:**
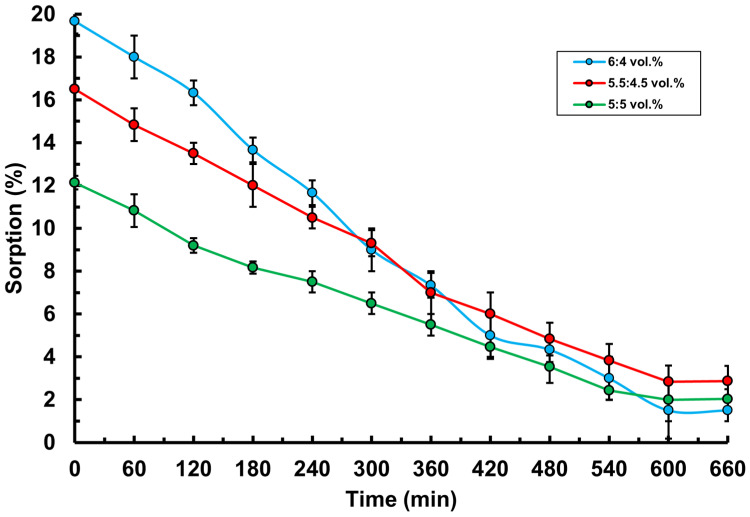
Sorption kinetics of the active pharmaceutical ingredient (ceftriaxone) by PVA-MC-based hydrogels: φ_PVA_:φ_MC_ = 6:4; 5.5:4.5; 5:5 vol.%.

The sorption (immobilization) of ceftriaxone into the hydrogels was studied using UV spectroscopy at a wavelength of 293 nm, which corresponds to the absorption peak of ceftriaxone. This characteristic wavelength allows for accurate measurement of the ceftriaxone concentration in the solution, providing high sensitivity and analytical precision. The optimal choice of wavelength ensures that the spectrophotometric method effectively detects absorption changes related to the presence of ceftriaxone, which is critical for studying its sorption in the hydrogel. The results showed that the percentage of ceftriaxone sorption decreased over time in all hydrogel ratios (Figure 7). However, at a higher PVA content of φ_PVA_:φ_MC_ = 6:4 vol.%, a more pronounced absorption of the drug was observed. Thus, it can be concluded that the hydrogel sample with a higher PVA content exhibits a more pronounced sorption capacity. This is attributed to the high hydrophilicity of PVA, which actively interacts with water and other polar solvents, forming numerous hydrogen bonds. These bonds increase the free space within the hydrogel structure, promoting more effective absorption and retention of the drug molecules. In addition, a high PVA concentration contributes to the formation of a dense cross-linked network, which enhances the sorption properties of the material.

**Figure 8:**
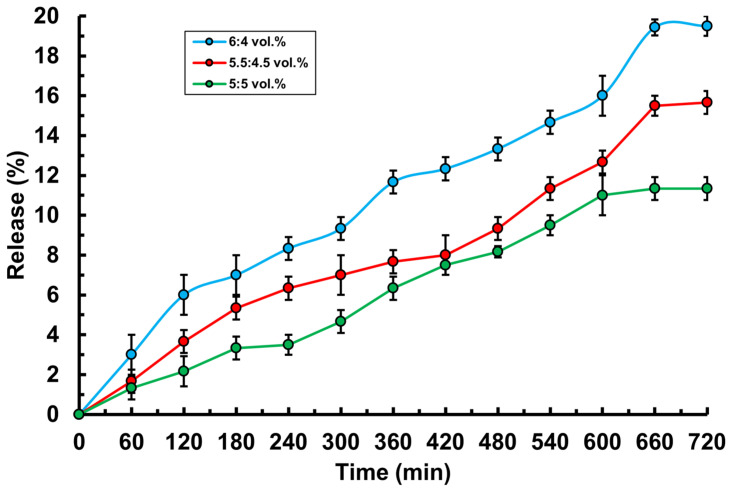
Release kinetics of the active pharmaceutical ingredient (ceftriaxone) from PVA-MC-based hydrogels: φ_PVA_:φ_MC_ = 6:4; 5.5:4.5; 5:5 vol.%.

To achieve a prolonged release effect, the drug embedded in the hydrogel must have a slow-release profile. To evaluate the release time, the release kinetics of ceftriaxone from the hydrogel were studied in isotonic solution (0.9% NaCl) (Figure 8). The results showed that ceftriaxone is released gradually under the influence of low-molecular-weight ions. The most optimal release—reaching nearly 20%—was observed in hydrogels with significantly high PVA content. Based on the obtained data, it can be concluded that ceftriaxone release takes a long time in all hydrogel compositions, which may be due to several factors. Hydrogels with a high degree of cross-linking and a dense network structure can significantly restrict the diffusion of the drug, making the movement of ceftriaxone molecules through the polymer matrix more difficult and leading to slower release. The high hydrophilicity of PVA and MC polymer components promotes the formation of strong hydrogen bonds with ceftriaxone molecules, which also slows down release, as the drug is tightly held within the hydrogel matrix. Additionally, ceftriaxone has a relatively large molecular size and specific charge, which further hinders its movement through the dense polymer matrix. As a result, all hydrogels demonstrated relatively gradual drug release, confirming their potential for use in prolonged drug delivery systems.

### 3.3. Microbiological Testing of PVA-MC-Based Hydrogels Containing Ceftriaxone

**Figure 9:**
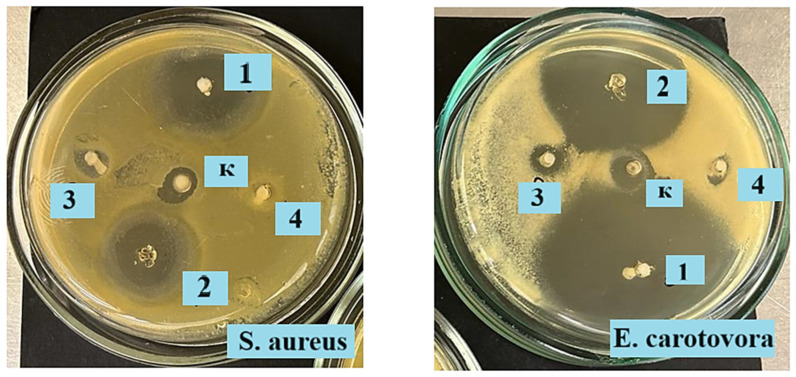
Inhibition zones of hydrogels against Gram-positive *S. aureus* and Gram-negative *E. carotovora*: φ_PVA_:φ_MC_/ceftriaxone = 6:4 (1), 5.5:4.5 (2), and 5:5 vol.% (3); φ_PVA_:φ_MC_ = 6:4 vol.% (4); Metrogyl Denta 1% (к).

Standard inoculation and cultivation methods were used during the study to determine the ability of hydrogels to inhibit the growth of various microorganisms. The antibacterial activity of PVA-MC hydrogel samples loaded with sodium ceftriaxone and the 1% Metrogyl Denta control was evaluated against a panel of Gram-positive and Gram-negative microorganisms using the disk diffusion method on solid nutrient media. The loaded hydrogels were tested against two bacterial strains, including *S. aureus* and *E. carotovora*. As shown in Table 1, the hydrogel sample with φ_PVA_:φ_MC_ = 6:4 vol.% (1) (loaded with ceftriaxone) exhibited a significant inhibition zone against the Gram-negative bacteria *E. carotovora* compared to the Gram-positive *S. aureus*. The inhibition zones for φ_PVA_:φ_MC_ = 6:4 vol.% (1) were 19 mm for *S. aureus* and 25 mm for *E. carotovora*, whereas for φ_PVA_:φ_MC_ = 5.5:4.5 vol.% (2), these values were slightly lower at 15 mm and 20 mm, respectively. In the case of φ_PVA_:φ_MC_ = 5:5 vol.% (3), the lowest inhibition zones were observed, measuring 4 mm and 5 mm, respectively. This may be attributed to the fact that, in this particular hydrogel ratio, the drug release kinetics study showed that the lowest amount of ceftriaxone was released over time compared to other ratios, and the concentration of the active substance did not reach the level required to effectively eliminate a greater number of bacteria. Meanwhile, the PVA-MC hydrogel without ceftriaxone showed no significant bactericidal effect. The inhibition zones for the 1% Metrogyl Denta control sample were 5 mm and 7 mm against *S. aureus* and *E. carotovora*, respectively. The antibacterial activity of the hydrogels φ_PVA_:φ_MC_ = 6:4 vol.% (1) and φ_PVA_:φ_MC_ = 5.5:4.5 vol.% (2) (loaded with sodium ceftriaxone) was expectedly higher than that of 1% Metrogyl Denta, indicating a promising antibacterial potential of PVA-MC mixtures loaded with ceftriaxone (Figure 9).

The results demonstrated that hydrogels containing ceftriaxone effectively inhibited bacterial growth, showing potential for use as antibacterial agents. This experiment confirms the feasibility of applying such hydrogels for the treatment of infected wounds and other biomedical applications where prolonged antibacterial action is required.

### 3.4. Determination of Mucoadhesive Properties of PVA-MC-Based Hydrogels Containing Ceftriaxone

Mucoadhesion is the process of interaction and adhesion of a polymer material to the mucous membrane, which lines the internal surfaces of organs such as the oral cavity, gastrointestinal tract, nasal cavity, eyes, and vagina. The mucosal membrane consists of a layer of epithelial cells covered with mucus containing glycoproteins and water. Adhesion of the polymer to the mucosa is achieved through physicochemical interactions such as hydrogen bonding, ionic interactions, and van der Waals forces [38]. The mucoadhesive properties of hydrogels play an important role in the development of effective drug delivery systems, especially for delivery through mucosal surfaces. Buccal drug delivery systems represent a method of administering drugs through the cheek mucosa. This route allows the drug to be rapidly absorbed into the bloodstream, bypassing the gastrointestinal tract and the liver, which can improve bioavailability and reduce metabolic losses.

**Figure 10:**
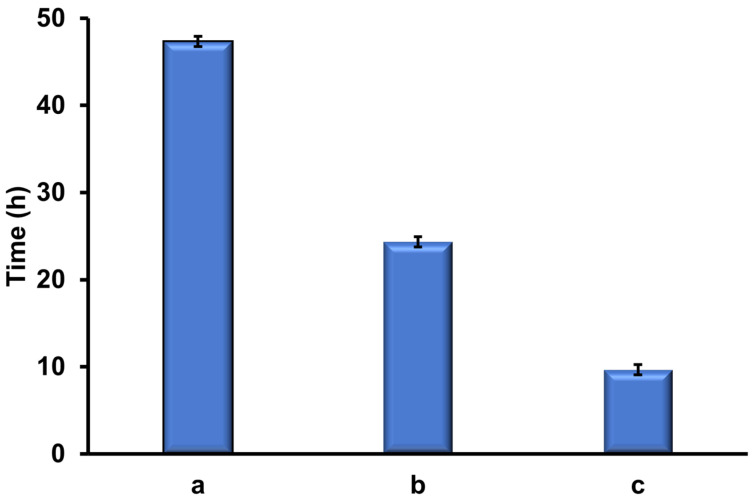
Duration of adhesion of PVA-MC-based hydrogels on the mucosal surface: φ_PVA_:φ_MC_ = 6:4 (a); 5.5:4.5 (b); 5:5 vol.% (c).

**Figure 11:**
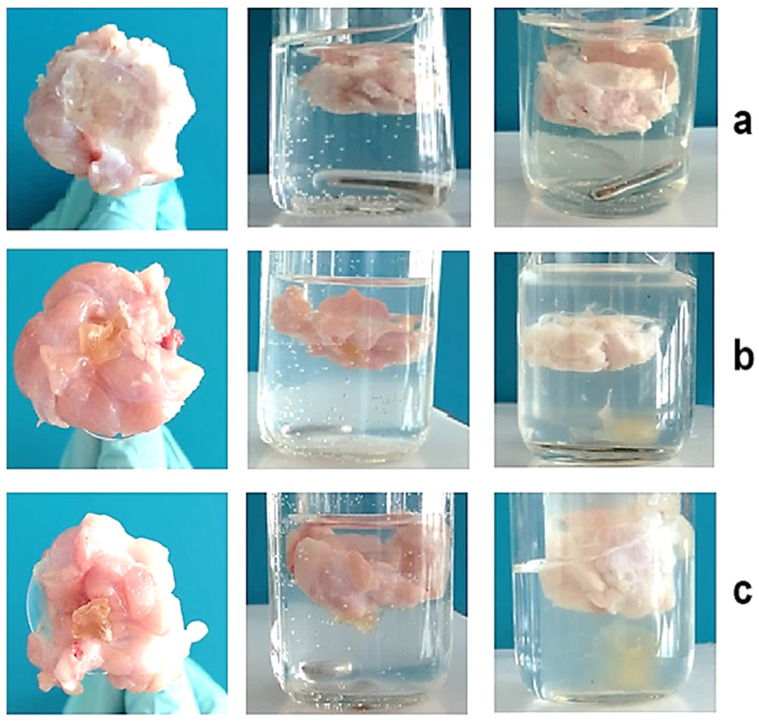
Investigation of the mucoadhesive properties of PVA-MC-based hydrogels: φ_PVA_:φ_MC_ = 6:4 (**a**); 5.5:4.5 (**b**); 5:5 vol.% (**c**).

In this study, the mucoadhesive properties of PVA-MC-based hydrogels were investigated. To study these properties, the rotating basket method was used. This method involves placing the hydrogel sample into a basket that rotates in a liquid medium simulating physiological conditions. During the experiment, the adhesion of the hydrogel to the mucosal surface is evaluated by determining its ability to stick and remain on the surface. This method allows for the assessment of the strength and stability of mucoadhesive materials under conditions close to those found in the body. Experiments were conducted using fresh porcine cheeks and dried hydrogel samples. The PVA-MC hydrogel with a volume ratio of 6:4 vol.% remained adhered to the surface for two days after immersion in artificial saliva, while the hydrogel with a ratio of 5.5:4.5 vol.% detached after one day. The third hydrogel sample adhered to the mucosal surface for approximately 10 h (Figure 10). As shown in Figure 11, all samples demonstrate good adhesion, but they exhibit different behaviors in artificial saliva solution.

## 4. Conclusions

Mixtures containing PVA and MC were obtained, based on which hydrogels were synthesized. It was established that the physicochemical properties of the hydrogels depend on the variation in the volume fraction of PVA in the mixture. FTIR spectroscopic analysis of the synthesized hydrogels was carried out. The swelling kinetics of PVA-MC-based hydrogels were studied in water and artificial saliva. It was found that the equilibrium swelling degree in artificial saliva is nearly twice as high as in water. It was also observed that increasing the volumetric content of PVA leads to an increase in the swelling degree. The sorption and release kinetics of the active pharmaceutical ingredient from the hydrogels were studied. It was shown that hydrogels with a higher PVA content exhibit greater sorption capacity. However, with increased PVA content, the release of the drug occurs at a slower rate.

The antibacterial properties of PVA-MC/ceftriaxone-based hydrogels were investigated. It was demonstrated that such hydrogels effectively inhibit bacterial growth, confirming their potential as antibacterial agents.

The mucoadhesive properties of PVA-MC-based hydrogels were also studied. It was established that the hydrogel with a PVA-MC ratio of 6:4 vol.% remained adhered to the mucosal surface for two days when immersed in saliva, while the hydrogel with a 5.5:4.5 vol.% ratio detached after one day. The third hydrogel sample adhered to the mucosal surface for approximately 10 h.

## Figures and Tables

**Table 1:** Results of the study on bactericidal activity against *S. aureus* and *E. carotovora*.

Sample Designation	Inhibition Zones, mm	
	*S. aureus*	*E. carotovora*
φ_PVA_:φ_MC_/ceftriaxone = 6:4 vol.%	19	25
φ_PVA_:φ_MC_/ceftriaxone = 5.5:4.5 vol.%	15	20
φ_PVA_:φ_MC_/ceftriaxone = 5:5 vol.%	4	5
φ_PVA_:φ_MC_ = 6:4 vol.% (without drug)	0	0
Metrogyl Denta 1% (control)	5	7

## Data Availability

Data will be available upon request.
